# Supplementary material for: Marked irregular myofiber shape is a hallmark of human skeletal muscle ageing and is reversed by heavy resistance training
Source: J Cachexia Sarcopenia Muscle. 2023 Dec 20;15(1):306–18. doi: 10.1002/jcsm.13405 (PMC10834339; doi:10.1002/jcsm.13405)

SM 1

	Young women (12)			Old women (13)			Oldest Old women (22)		
	Average ± SD	Min - Max	n	Average ± SD	Min - Max	n	Average ± SD	Min - Max	n
Age (y.)	23 ± 3	20 - 28	12	74 ± 3	71 - 78	13	87 ± 3	83 - 97	22
Height (m.)	1.68 ± 0.07	1.57 - 1.77	12	1.67 ± 0.03	1.62 - 1.74	13	1.59 ± 0.08	1.42 - 1.76	13
Weight (kg.)	64 ± 8	53 - 75	12	67 ± 9	57 - 84	13	57 ± 10	45 - 76	13
BMI (kg/m^2)	22.7 ± 2.0	19.0 - 25.5	12	24.2 ± 3.8	19.9 - 30.4	13	22.3 ± 2.9	18.7 - 26.2	13
LBM_{leg} (kg.)		N/A		13 ± 1	12 - 13	2	12 ± 2	9 - 17	12
CSA_{quad} (cm^2)		N/A			N/A		35 ± 8	26 - 48	10
Isometric MVC (Nm)		N/A			N/A		80 ± 31	22 - 140	10
Isokinetic MVC (Nm)		N/A			N/A		58 ± 19	18 - 91	10
	**Young men (22)**			**Old men (98)**			**Oldest Old men (30)**		
	Average ± SD	Min - Max	n	Average ± SD	Min - Max	n	Average ± SD	Min - Max	n
Age (y.)	26 ± 5	20 - 36	22	71 ± 4	60 - 79	98	86 ± 3	81 - 93	30
Height (m.)	1.81 ± 0.08	1.68 - 1.93	22	1.78 ± 0.07	1.55 - 1.95	98	1.76 ± 0.07	1.60 - 1.88	27
Weight (kg.)	82 ± 15	62 - 111	22	82 ± 12	56 - 109	98	79 ± 9	65 - 98	27
BMI (kg/m^2)	24.6 ± 3.3	20.1 - 30.4	22	25.9 ± 3.2	18.9 - 32.5	98	25.6 ± 3.0	20.7 - 33.2	27
LBM_{leg} (kg.)	22 ± 4	17 - 30	22	20 ± 3	13 - 26	51	18 ± 2	15 - 21	21
CSA_{quad} (cm^2)	73 ± 15	55 - 99	7	62 ± 12	36 - 91	65	50 ± 8	37 - 69	22
Isometric MVC (Nm)	272 ± 62	186 - 364	22	188 ± 38	85 - 278	93	145 ± 30	75 - 205	24
Isokinetic MVC (Nm)	307 ± 67	200 - 425	22	207 ± 46	75 - 313	94	130 ± 34	40 - 219	25

SM 2

	Soendenbroe et al., 2022 (n=46)			
	Old (n=31)		Young (n=15)	
	Average ± SD	Min - Max	Average ± SD	Min - Max
Age (yrs)	73 ± 4	68 - 82	26 ± 5	20 - 36
Height (m)	1.77 ± 0.07	1.61 - 1.95	1.83 ± 0.07	1.69 - 1.93
Weight (kg)	79 ± 10	63 - 109	82 ± 13	62 - 105
BMI (kg/m^2)	25.1 ± 3.3	20.5 - 32.4	24.5 ± 3.0	20.3 - 29.7
LBM_{leg} (kg)	20 ± 2	17 - 26	23 ± 3	18 - 27
Isometric MVC (Nm)	186 ± 38	118 - 278	285 ± 66	186 - 364
Isokinetic MVC (Nm)	197 ± 45	123 - 313	318 ± 74	200 - 425

	Karlsen et al., 2020a (n=26)			
	Old (n=19)		Young (n=7)	
	Average ± SD	Min - Max	Average ± SD	Min - Max
Age (yrs)	67 ± 4	60 - 73	25 ± 3	20 - 31
Height (m)	1.81 ± 0.07	1.68 - 1.94	1.79 ± 0.08	1.68 - 1.91
Weight (kg)	85 ± 14	60 - 108	81 ± 19	62 - 111
BMI (kg/m^2)	25.9 ± 3.6	18.9 - 32.5	25.0 ± 4.2	20.1 - 30.4
LBM_{leg} (kg)	20 ± 3	15 - 26	21 ± 5	17 - 30
CSA_{quad} (cm^2)	58 ± 11	36 - 82	73 ± 15	55 - 99
Isometric MVC (Nm)	200 ± 36	130 - 257	244 ± 45	203 - 334
Isokinetic MVC (Nm)	230 ± 42	143 - 295	284 ± 44	230 - 358

	Heisterberg et al., 2018 (n=52)	
	Average ± SD	Min - Max
Age (yrs)	72 ± 5	65 - 85
Height (m)	1.78 ± 0.07	1.61 - 1.90
Weight (kg)	84 ± 11	57 - 108
BMI (kg/m^2)	26.5 ± 3.0	19.2 - 33.2
CSA_{quad} (cm^2)	62 ± 11	37 - 91
Isometric MVC (Nm)	177 ± 38	85 - 256
Isokinetic MVC (Nm)	197 ± 47	75 - 298

	Bechshøft et al., 2019 (n=23)			
	Old (n=11)		Young (n=12)	
	Average ± SD	Min - Max	Average ± SD	Min - Max
Age (yrs)	74 ± 3	71 - 78	23 ± 3	20 - 28
Height (m)	1.66 ± 0.03	1.62 - 1.69	1.68 ± 0.07	1.57 - 1.77
Weight (kg)	69 ± 10	57 - 84	64 ± 8	53 - 75
BMI (kg/m^2)	25.0 ± 3.6	20.3 - 30.4	22.7 ± 2.0	19.0 - 25.5

	Bechshøft et al., 2017 (n=29)	
	Average ± SD	Min - Max
Age (yrs)	87 ± 3	83 - 94
Height (m)	1.69 ± 0.11	1.42 - 1.88
Weight (kg)	71 ± 13	45 - 98
BMI (kg/m^2)	24.7 ± 3.1	19.0 - 30.8
LBM_{leg} (kg)	16 ± 3	9 - 21
CSA_{quad} (cm^2)	43 ± 10	26 - 69
Isometric MVC (Nm)	120 ± 45	22 - 205
Isokinetic MVC (Nm)	94 ± 37	18 - 148

	Karlsen et al., 2020b (n=9)	
	Average ± SD	Min - Max
Age (yrs)	77 ± 7	69 - 91
Height (m)	1.70 ± 0.09	1.55 - 1.83
Weight (kg)	64 ± 11	48 - 85
BMI (kg/m^2)	22.2 ± 3.2	18.7 - 29.1
LBM_{leg} (kg)	13 ± 2	10 - 15

	Kryger & Andersen, 2007 (n=12)	
	Average ± SD	Min - Max
Age (yrs)	88 ± 3	85 - 97

SM 3

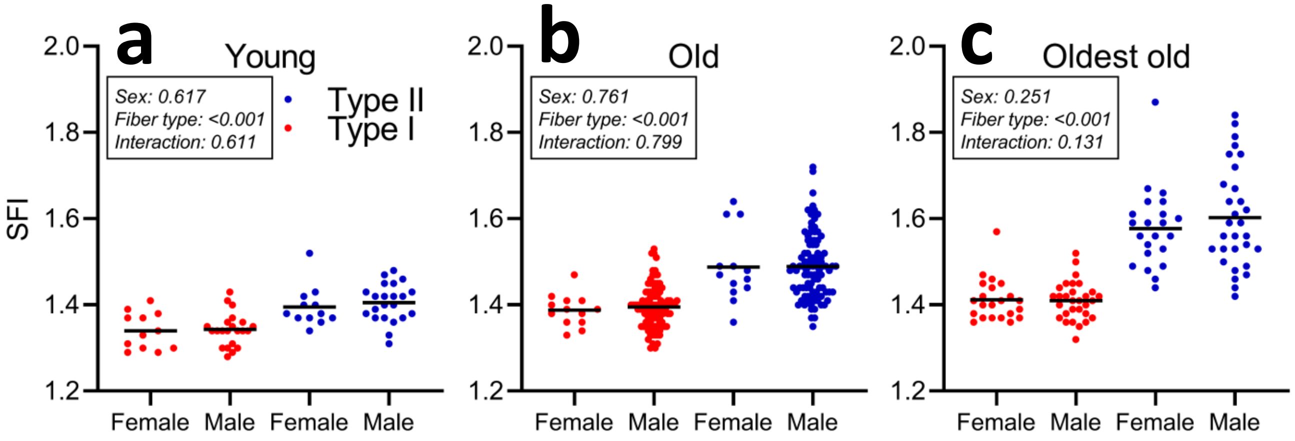
a
Young
b
Old
c
Oldest old
SFI
2.0
1.8
1.6
1.4
1.2
Sex: 0.617
Fiber type: <0.001
Interaction: 0.611
Type II
Type I
Sex: 0.761
Fiber type: <0.001
Interaction: 0.799
Sex: 0.251
Fiber type: <0.001
Interaction: 0.131
Female
Male
Female
Male

SM 4

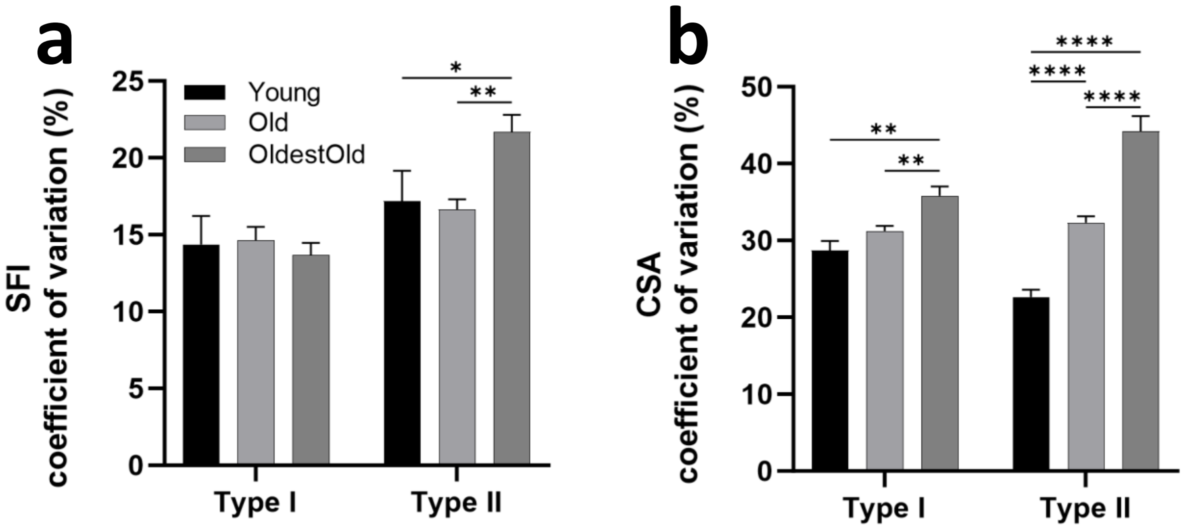

a
SFI
coefficient of variation (%)
25
20
15
10
5
0
Young
Old
OldestOld
*
**
Type I
Type II
b
CSA
coefficient of variation (%)
50
40
30
20
10
0
**
**
****
****
****
Type I
Type II

SM 5

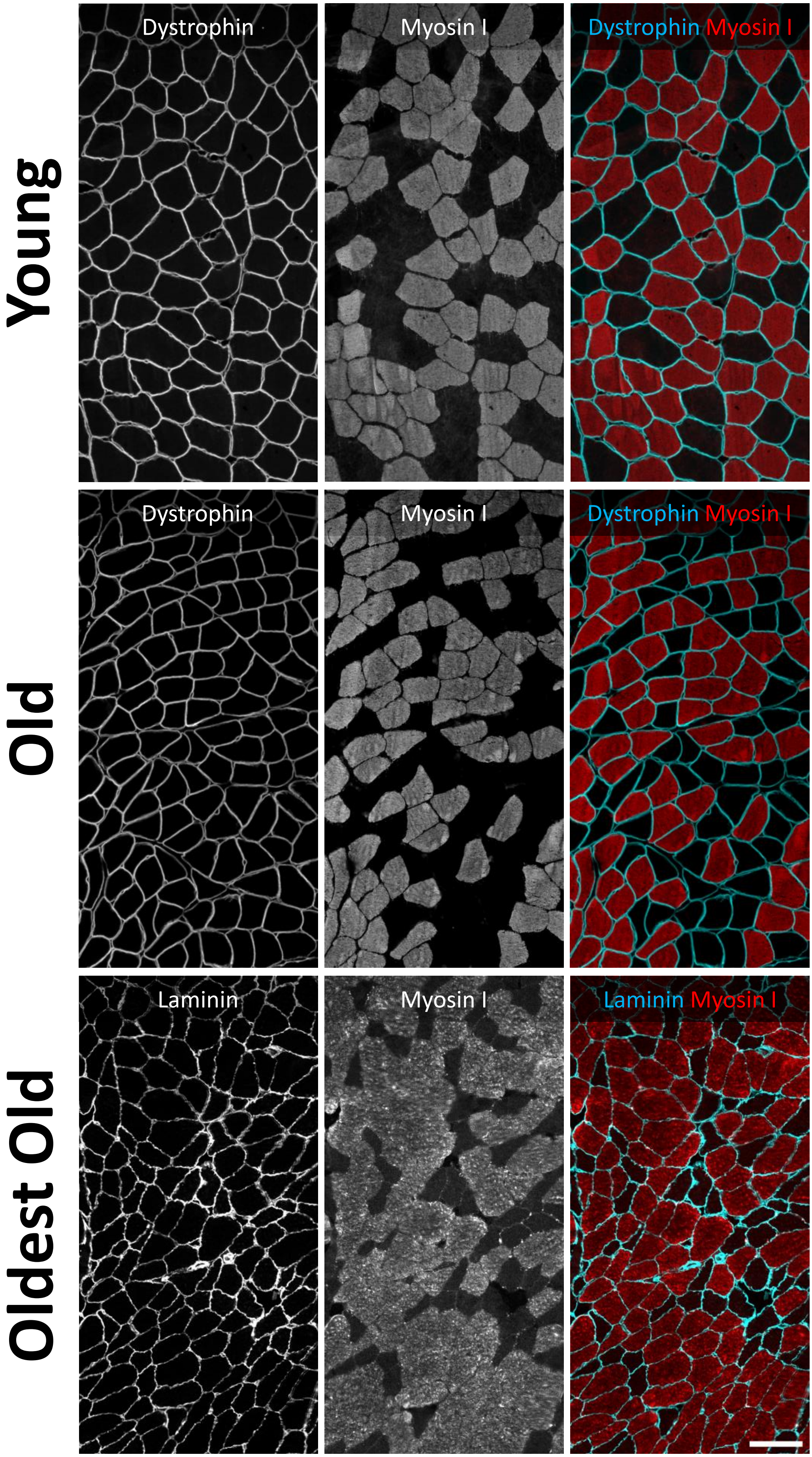
Young
Old
Oldest Old
Dystrophin
Myosin I
Dystrophin Myosin I
Dystrophin
Myosin I
Dystrophin Myosin I
Laminin
Myosin I
Laminin Myosin I

SM 6

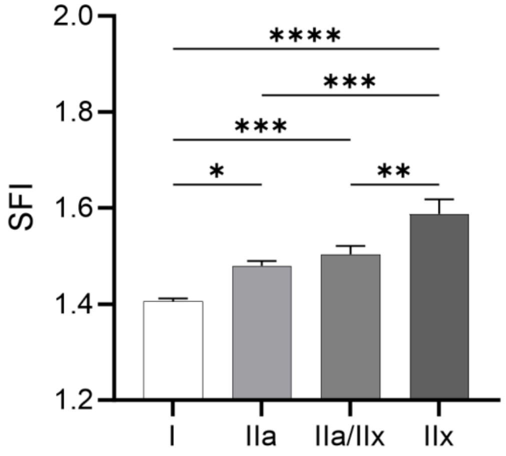

SM 7

a

		Number of subjects in each SFI increment			Number of myofibers in each SFI increment		
		Young	Old	Oldest Old	Young	Old	Oldest Old
Type I	<.1.10	1	1	1	1	1	1
	1.10-1.20	34	99	49	497	992	316
	1.20-1.30	34	111	52	2859	5916	2264
	1.30-1.40	34	111	52	2134	6041	2663
	1.40-1.50	34	111	52	984	3576	1760
	1.50-1.60	34	107	51	421	1845	977
	1.60-1.70	30	101	52	144	946	505
	1.70-1.80	21	93	45	85	464	290
	1.80-1.90	14	76	41	33	253	161
	1.90-2.00	7	56	34	10	128	103
	>2.00	16	67	40	35	233	184
Type II	<.1.10	0	0	1	0	0	1
	1.10-1.20	29	79	34	191	410	142
	1.20-1.30	34	111	52	1980	2924	1012
	1.30-1.40	34	111	52	2310	4303	1705
	1.40-1.50	34	111	52	1326	3266	1495
	1.50-1.60	34	111	52	673	2272	1154
	1.60-1.70	33	108	52	306	1287	774
	1.70-1.80	31	104	51	161	912	512
	1.80-1.90	27	96	50	75	537	385
	1.90-2.00	15	88	51	28	336	238
	>2.00	26	95	51	68	776	757

b

		Number of subjects in each CSA increment			Number of myofibers in each CSA increment		
		Young	Old	Oldest Old	Young	Old	Oldest Old
Type I	0-1000	10	37	31	73	188	178
	1000-2000	23	78	49	423	1126	647
	2000-3000	33	99	52	1256	3574	1447
	3000-4000	34	110	51	1692	4641	2015
	4000-5000	34	110	49	1526	4421	1812
	5000-6000	31	109	45	1068	3187	1413
	>6000	29	103	41	1166	3407	1734
Type II	0-1000	7	52	46	35	923	1675
	1000-2000	16	92	50	313	2803	2707
	2000-3000	27	107	50	898	4548	1824
	3000-4000	33	110	48	1447	4198	1048
	4000-5000	33	105	41	1634	2418	517
	5000-6000	29	96	27	1422	1132	234
	>6000	22	66	17	1371	1018	187

SM 8

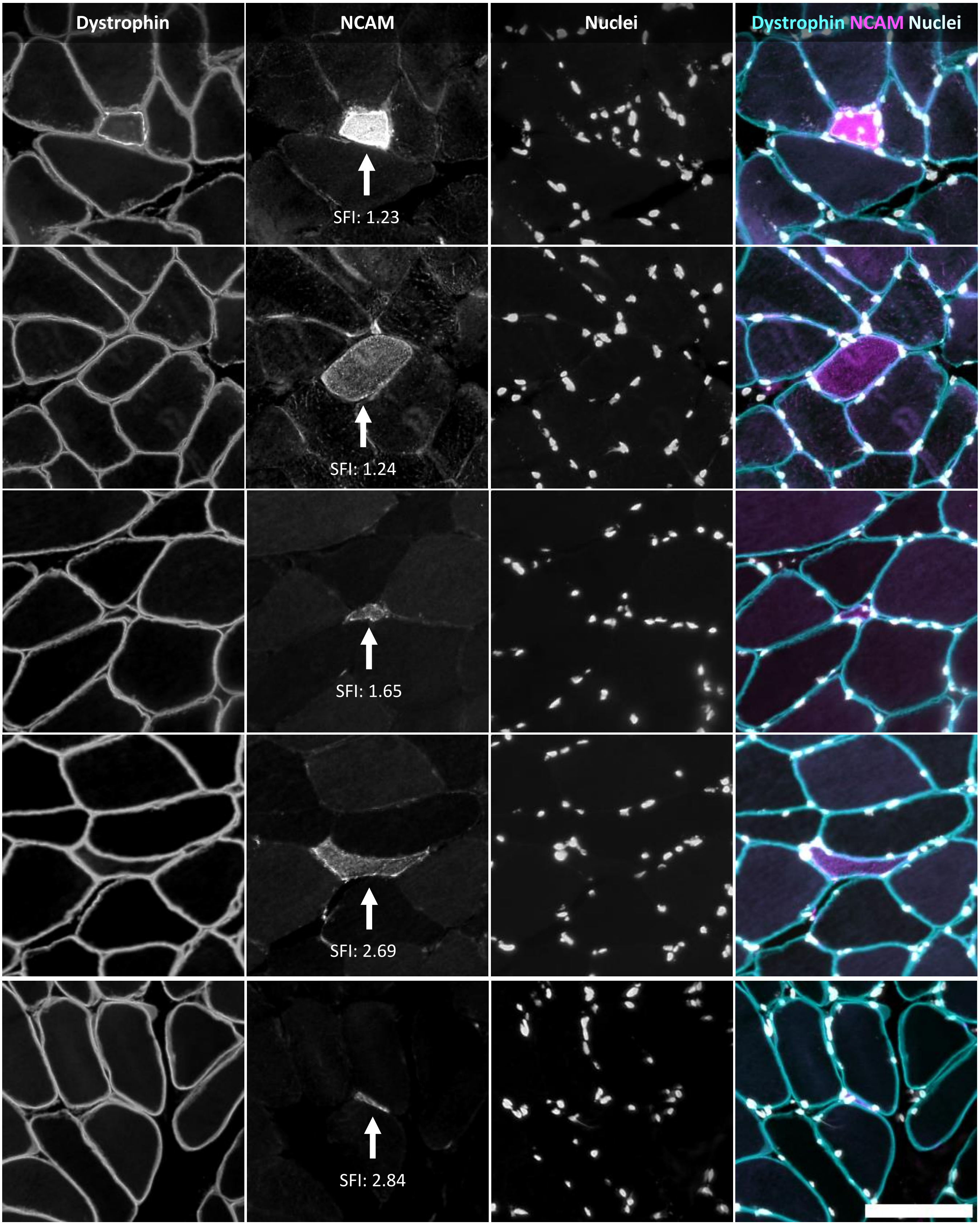
Dystrophin
NCAM
Nuclei
Dystrophin NCAM Nuclei
SFI: 1.23
SFI: 1.24
SFI: 1.65
SFI: 2.69
SFI: 2.84

SM 9

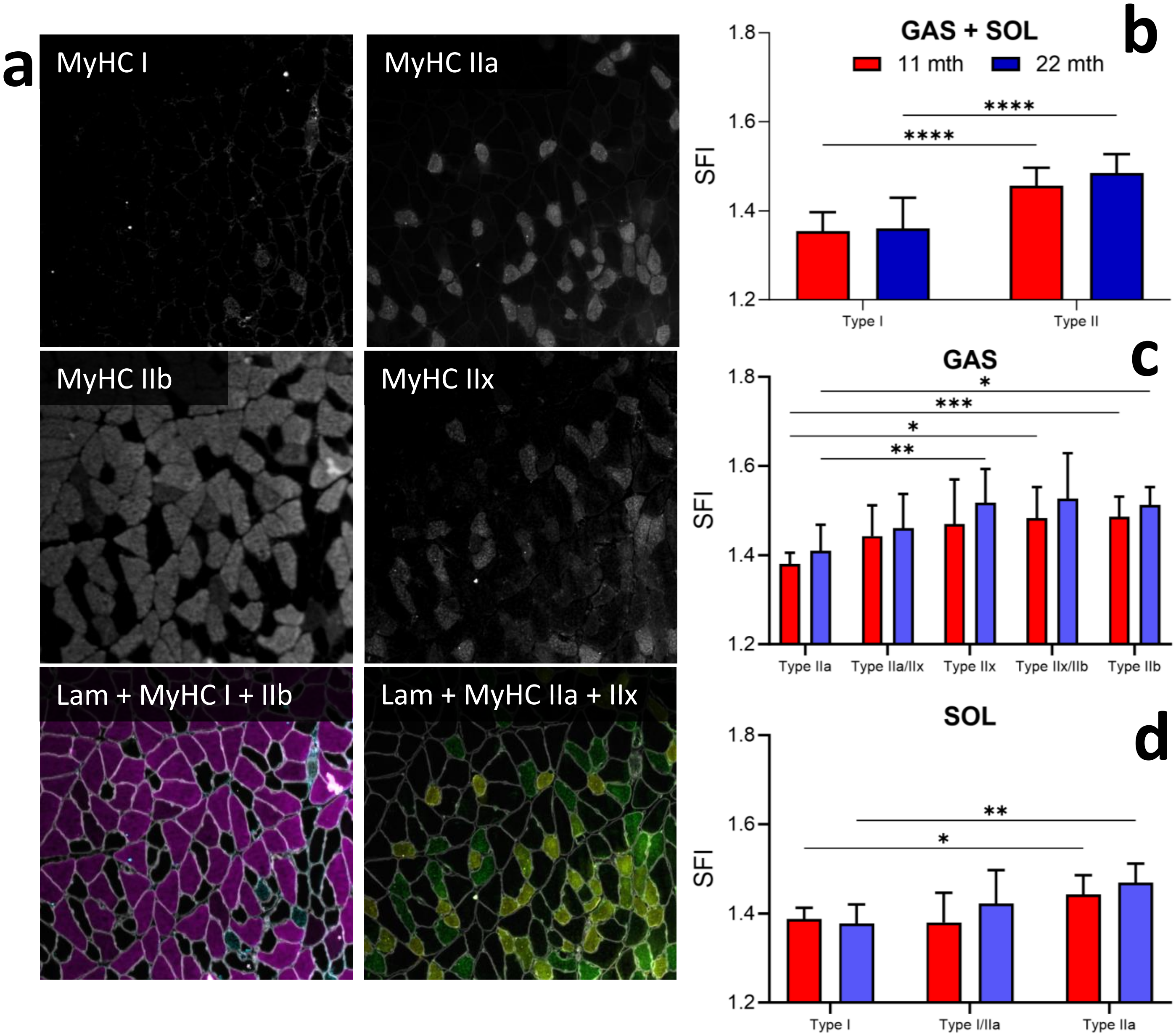

a
MyHC I
MyHC IIa
MyHC IIb
MyHC IIx
Lam + MyHC I + IIb
Lam + MyHC IIa + IIx
b
GAS + SOL
11 mth
22 mth
SFI
1.8
1.6
1.4
1.2
****
****
Type I
Type II
c
GAS
SFI
*
***
*
**
Type IIa
Type IIa/IIx
Type IIx
Type IIx/IIb
Type IIb
d
SOL
SFI
**
*
Type I
Type I/IIa
Type IIa

SM 10

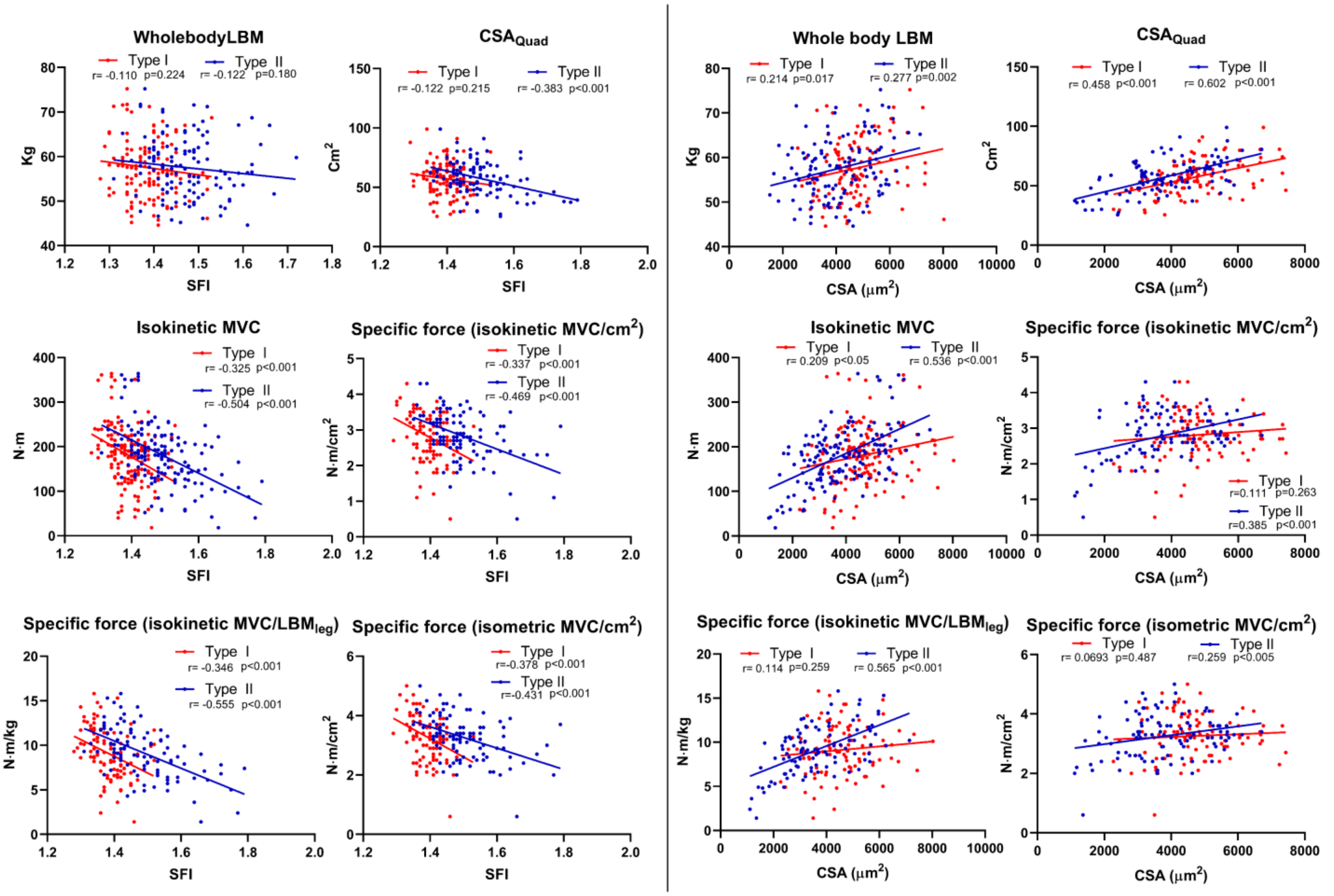

WholebodyLBM
Type I r= -0.110 p=0.224
Type II r= -0.122 p=0.180
CSAQuad
Type I r= -0.122 p=0.215
Type II r= -0.383 p<0.001
Whole body LBM
Type I r= 0.214 p=0.017
Type II r= 0.277 p=0.002
CSAQuad
Type I r= 0.458 p<0.001
Type II r= 0.602 p<0.001
Isokinetic MVC
Type I r= -0.325 p<0.001
Type II r= -0.504 p<0.001
Specific force (isokinetic MVC/cm2)
Type I r= -0.337 p<0.001
Type II r= -0.469 p<0.001
Isokinetic MVC
Type I r= 0.209 p<0.05
Type II r= 0.536 p<0.001
Specific force (isokinetic MVC/cm2)
Type I r=0.111 p=0.263
Type II r=0.385 p<0.001
Specific force (isokinetic MVC/LBMleg)
Type I r= -0.346 p<0.001
Type II r= -0.555 p<0.001
Specific force (isometric MVC/cm2)
Type I r=-0.378 p<0.001
Type II r=-0.431 p<0.001
Specific force (isokinetic MVC/LBMleg)
Type I r= 0.114 p=0.259
Type II r= 0.565 p<0.001
Specific force (isometric MVC/cm2)
Type I r= 0.0693 p=0.487
Type II r=0.259 p<0.005
Kg
Cm2
N·m
N·m/cm2
N·m/kg
SFI
CSA (μm2)

SM 11

LBMleg

Resulting model: n=108, p<0.0001, R Squared=41%

	Coefficient	SE	p-value	R Squared
Intercept	32,79	8,61	0,0002	
Type I SFI	-14,23	5,97	0,0190	0,032
Type II CSA	0,002	0,0002	<0,0001	0,272

Isometric MVC

Resulting model: n=150, p<0.0001, R Squared=58%

	Coefficient	SE	p-value	R Squared
Intercept	579,61	69,55	<0,0001	
Age	-2,25	0,25	<0,0001	0,229
Type II SFI	-187,70	53,09	0,0005	0,036
Type I CSA	0,01	0,004	0,0013	0,031

Isometric RFD

Resulting model: n=123, p<0.0001, R Squared=35%

	Coefficient	SE	p-value	R Squared
Intercept	2847,71	182,97	<0,0001	
Age	-22,23	2,78	<0,0001	0,346

Specific force (isometric MVC/LBMleg)

Resulting model: n=98, p<0.0001, R Squared=57%

	Coefficient	SE	p-value	R Squared
Intercept	23,99	3,23	<0,0001	
Age	-0,08	0,01	<0,0001	0,270
Type II SFI	-5,49	2,40	0,0242	0,024

Whole body LBM

Resulting model: n=123, p=0.0022, R Squared=8%

	Coefficient	SE	p-value	R Squared
Intercept	51,26	2,08	<0,0001	
Type II CSA	0,002	0,0005	0,0022	0,075

CSAquad

Resulting model: n=104, p<0.0001, R Squared=55%

	Coefficient	SE	p-value	R Squared
Intercept	98,32	19,64	<0,0001	
Age	-0,26	0,08	0,0016	0,048
Type II SFI	-36,30	14,00	0,0110	0,030
Type I CSA	0,004	0,001	0,0002	0,068
Type II CSA	0,003	0,001	0,0081	0,033

Isokinetic MVC

Resulting model: n=150, p<0.0001, R Squared=57%

	Coefficient	SE	p-value	R Squared
Intercept	516,30	64,08	<0,0001	
Age	-2,04	0,23	<0,0001	0,231
Type II SFI	-164,82	48,84	0,0009	0,034
Type I CSA	0,01	0,003	0,0024	0,028

Specific force (isokinetic MVC/LBMleg)

Resulting model: n=99, p<0.0001, R Squared=57%

	Coefficient	SE	p-value	R Squared
Intercept	22,13	3,06	<0,0001	
Age	-0,08	0,01	<0,0001	0,264
Type II SFI	-5,33	2,27	0,0209	0,025

Specific force (isokinetic MVC/CSAquad)

Resulting model: n=103, p<0.0001, R Squared=34%

	Coefficient	SE	p-value	R Squared
Intercept	9,78	1,72	<0,0001	
Type I SFI	-3,60	1,47	0,0164	0,040
Type II SFI	-1,79	0,80	0,0273	0,034
Type II CSA	0,0002	0,00005	0,0002	0,100

Specific force (isometric MVC/CSAquad)

Resulting model: n=103, p<0.0001, R Squared=26%

	Coefficient	SE	p-value	R Squared
Intercept	7,79	1,11	<0,0001	
Age	-0,01	0,00	0,0031	0,068
Type II SFI	-2,33	0,83	0,0060	0,059

SM 12

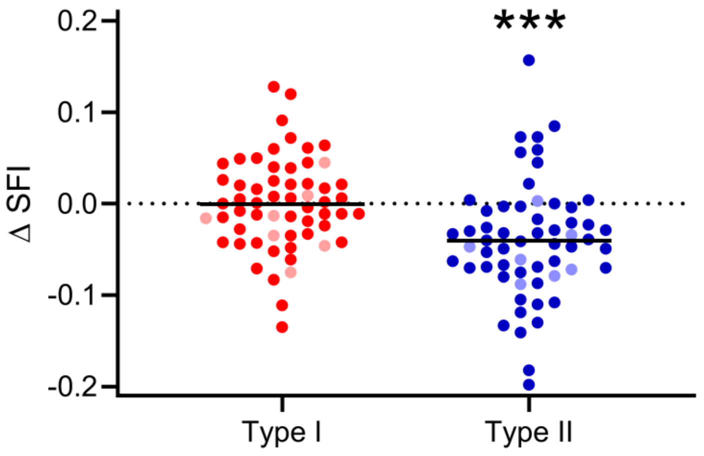

Supplement: Supplementary file 1 — Supporting Information S1. Subject characteristics for males and females separately, shown as means ± SD with range and number of subjects represented in each measurement. BMI, body mass index. LBMleg, leg lean mass. CSAquad, quadriceps cross‐sectional area. MVC, maximal voluntary contraction. Supporting Information S2. Subject characteristics for each of the original studies separately, shown as means ± SD with minimum and maximum values. These subjects characteristics have previously been reported in the original publications13,24–29. BMI, body mass index. LBMleg, leg lean mass. CSAquad, quadriceps cross‐sectional area. MVC, maximal voluntary contraction. Supporting Information S3. SFI in males and females separately. Type I (red) and II (blue) myofiber SFI for Young (a, n = 22 males and 12 females), Old (b, n = 98 males and 13 females), and Oldest Old (c, n = 30 males and 22 females). Data were analyzed using Two‐Way Repeated Measures ANOVA (sex x fiber type) within each group, with main effects of fiber type indicated in the figure. SFI, shape factor index. Supporting Information S4. Coefficient of variation (CV) of SFI (A) and CSA (B) for type I and II myofibers in Young (n = 34), Old (n = 111) and Oldest Old (n = 52). Data were analyzed using Two‐Way Repeated Measures ANOVA (age group x fiber type). *p < 0.05, **p < 0.01, ****p < 0.0001. CV, coefficient of variation. SFI, shape factor index. CSA, cross‐sectional area. Supporting Information S5. Representative split channel images of muscle biopsy cross‐sections from Young, Old, and Oldest Old, stained for dystrophin (Young, Old) or laminin (Oldest Old), and MyHC I. Scale bar = 100 μm. Supporting Information S6. SFI in hybrid fibers. The SFI of IIa/IIx hybrid fibers were evaluated in a subset of samples (n = 52). Data were analyzed using One‐Way Repeated Measures ANOVA (fiber type). *p < 0.05, **p < 0.005, ***p < 0.005, ****p < 0.0001. SFI, shape factor index. Supporting Information S7. a) Number of subjects an [file JCSM-15-306-s001.pdf]
