# Supplementary material for: Marked irregular myofiber shape is a hallmark of human skeletal muscle ageing and is reversed by heavy resistance training
Source: J Cachexia Sarcopenia Muscle. 2023 Dec 20;15(1):306–18. doi: 10.1002/jcsm.13405 (PMC10834339; doi:10.1002/jcsm.13405)
Supplement: Supplementary file 2 — Data S1. Supporting Information. [file JCSM-15-306-s002.docx]

**Supplemental references**

S1. Schindelin J, Arganda-Carreras I, Frise E, Kaynig V, Longair M, Pietzsch T et al. Fiji: an open-source platform for biological-image analysis. Nature Methods 2012;9:676–682.

S2. Ross L. The Image Processing Handbook , 7th ed., John C. Russ and F. Brent Neal. CRC Press, Boca Raton, FL, 2015, 1053 pp. ISBN: 978-1498740265. Microscopy and Microanalysis 2016;22:733.

S3. Roberts BM, Lavin KM, Many GM, Thalacker-Mercer A, Merritt EK, Bickel CS et al. Human neuromuscular aging: Sex differences revealed at the myocellular level. Exp Gerontol 2018;106:116–124

S4. von Haehling S, Coats AJS, Anker SD. Ethical guidelines for publishing in the Journal of Cachexia, Sarcopenia and Muscle: update 2021. J Cachexia Sarcopenia Muscle 2021;12:2259–2261.
